# Rapid crystallization during recycling of basaltic andesite tephra: timescales determined by reheating experiments

**DOI:** 10.1038/srep46364

**Published:** 2017-04-12

**Authors:** Nicholas Deardorff, Katharine Cashman

**Affiliations:** 1 Indiana University of Pennsylvania, Department of Geoscience, 302 East Walk, 111 Walsh Hall, Indiana, PA, 15701, USA; 2 University of Bristol, School of Earth Sciences, Queens Road, Bristol BS8 1RJ, United Kingdom

## Abstract

Microcrystalline inclusions within microlite-poor matrix are surprisingly common in low intensity eruptions around the world, yet their origin is poorly understood. Inclusions are commonly interpreted as evidence of crystallization along conduit margins. Alternatively, these clasts may be recycled from low level eruptions where they recrystallize by heating within the vent. We conducted a series of experiments heating basaltic andesite lapilli from temperatures below the glass transition (~690 °C) to above inferred eruption temperatures (>1150 °C) for durations of 2 to >60 minutes. At 690 °C < T < 800 °C, crystallization is evident after heating for ~20 minutes; at T > 800 °C, crystallization occurs in <5 minutes. At T ≥ 900 °C, all samples recrystallize extensively in 2–10 minutes, with pyroxenes, Fe-oxides, and plagioclase. Experimental crystallization textures closely resemble those observed in natural microcrystalline inclusions. Comparison of inclusion textures in lapilli from the active submarine volcano NW Rota-1, Mariana arc and subaerial volcano Stromboli suggest that characteristic signatures of clast recycling are different in the two environments. Specifically, chlorine assimilation provides key evidence of recycling in submarine samples, while bands of oxides bordering microcrystalline inclusions are unique to subaerial environments. Correct identification of recycling at basaltic vents will improve (lower) estimates of mass eruption rate and help to refine interpretations of eruption dynamics.

Tephra produced by explosive eruptions provides important information about magma ascent, vesiculation, fragmentation, and deposition. Mafic pyroclasts from strombolian to violent strombolian eruptions are characterized by a wide range in both vesicularity and groundmass crystallinity. In particular, mafic pyroclasts are often classified as either microlite-poor (sideromelane) or microlite-rich (tachylite)^1^. Both sideromelane and tachylite are often found within the same depositional layers, and even within a single clast. However, the origin of these clast types, and the ascent and eruption conditions implied by the variable proportions of different clasts types, is not well understood. Correct identification of microcrystalline textures has significant implications as these textures are often used to interpret eruption dynamics.

The presence of both sideromelane and tachylite is a common feature of tephra deposits from cinder cone eruptions (e.g., Cinder Cone, CA^1^; Stromboli, Italy^2^,^3^; Mt. Etna, Italy^4^; Parícutin, Mexico^5^; Newberry Volcano, OR^6^; Mt. Vesuvius, Italy^7^). Sideromelane clasts are generally assumed to represent primary (deeper) magma that ascends rapidly and erupts. Tachylite clasts have been interpreted as slow-moving magma incorporated from along conduit walls^3^,^4^ or as magma stored temporarily in shallow dikes and sills^5^,^8^. Both scenarios call upon tachylite-forming magma to have sufficiently long residence times within the upper crust to allow magma degassing and crystallization prior to eruption. An alternative explanation is additional residence time in the vent by recycling previously erupted clasts^9^,^10^, a mechanism that should be enhanced by mass eruption rates (MERs) sufficiently low that clasts are not transported far from the vent. An extreme example comes from mild submarine eruptions observed at NW Rota-1 (Mariana arc), where eruption plumes are suppressed by the overlying water column^9^. Abundant pyroclast recycling has also been described at Stromboli volcano (Italy)^11^. In both cases, the extent of vent clogging affects the subsequent eruption intensity^12^ and grain size distribution^9^. Importantly, clogged vents may promote reheating of the recycled clasts.

Textural evidence of recycling may include pyroclasts with additional groundmass crystallization, precipitation of sublimates on external surfaces, and changes in color, luster and external morphologies^10^,^13^. Recycled clasts may also form microcrystalline inclusions within more juvenile, less microcrystalline matrix that display varying degrees of deformation, mingling, and banding^9^ (Fig. 1). A common feature of the latter is an oxide-rich layer surrounding the included clast. In contrast recycling in the submarine environment may preserve geochemical evidence of seawater assimilation, specifically as chlorine enrichment within microcrystalline (recycled) inclusions^9^. The common component in recycled tephra, whether entrained in juvenile melt or not, is increased groundmass crystallization. However, the time required for such crystallization is not well-constrained. Here we constrain time scales of crystallization and recreate recycling microcrystalline textures by pyroclast reheating experiments. Identification of common recycling textures and how they vary with time, temperature, and oxidation conditions may allow us to determine signatures of recycling in natural samples.

Crystallization is usually studied as a cooling-driven phenomenon. However, heating glass above the glass transition temperature (but below the liquidus) can also cause crystallization^13^,^14^. The limited experimental data on heating-induced crystallization show that the crystallization kinetics are interface-controlled and depend on oxidation state as well as temperature, and that environments that promote such crystallization include overtopping lava flows in pahoehoe fields^14^ and intra-crater pyroclast accumulation^13^. Our experiments complement those of D’Oriano, *et al*.^13^ by exploring shorter timescales (2–64 min) and covering a larger temperature range (<690 °C–1170 °C). In this study we (1) establish textural criteria to recognize heating-induced crystallization in both subaerial and submarine environments and (2) constrain time scales of clast recycling. The implications for incorrectly identifying pyroclast recycling include potential overestimation of MER by misinterpreting recycled clasts as juvenile and misinterpretation of eruption dynamics and history of crystallization through microlite textures (e.g. crystallization via long residence time along conduit walls vs recycling).

## Methods

We conducted reheating experiments on natural basaltic andesite (~55 wt% SiO_2_) lapilli from Parícutin, Mexico to test the effect of reheating on microcrystallinity in tephra. We chose to use low crystallinity (sideromelane) Parícutin lapilli because the eruptions and deposits have been well studied^5^,^15^ and are of similar composition to NW Rota-1 (~52–53 wt% SiO_2_). We also attempted heating experiments on NW Rota-1 samples, however heating the clasts resulted in an odd alteration where the glass ‘inflated’ producing a bubbly, popcorn texture that was very brittle, and we were unable to polish or analyze the clasts. The ‘inflation’ of NW Rota-1 glass may be due to moderately hydrous glass (H_2_O: 0.3–1.1 wt%, determined through Fourier transform infrared spectroscopy). No inflation was observed in any of the heated Parícutin clasts. Additionally, microcrystalline inclusions are prevalent in NW Rota-1 samples, found in most sideromelane clasts examined, making them less desirable as experimental specimens. Microcrystalline inclusions have been observed in a few dark and dense (tachylite) Parícutin clasts (Ref. 16; clast types described in ref. 5) but were not present in any of the tan (sideromelane) clasts used in the reheating experiments.

Clasts 4–8 mm in diameter were split, with one half saved as a control and one half heated in a one-atmosphere Deltec vertical tube furnace at atmospheric oxygen fugacities (*f*O_2_). To constrain crystallization kinetics, we heated samples over different time intervals from room temperature to experimental temperatures ranging from below the glass transition temperature (T_g_) of basalt at ~690 °C^17^ to 1170 °C, which we infer to be above the eruption temperature and approaches the liquidus (~1178 °C; calculated in MELTS^18^,^19^). The tube furnace was brought up to temperature before each sample was inserted. 1D modeling using Fourier’s heat flow equation shows that the center of a solid 4 mm diameter sphere will equilibrate with the furnace temperature within approximately 30 seconds at all experimental temperatures considered in this study. These calculations provide a conservative estimate as all of the pyroclasts were vesicular. Upon completion of the experiment the samples were promptly removed to cool in air at room temperature. During removal from the furnace, clast temperature fell below the glass transition temperature within seconds. Heating times reported indicate the length of time the clast was in the furnace and above T_g_. Each clast was heated isothermally for 2 to 64 minutes at T = 620–1000 °C or 5–30 minutes at T ≥ 1100 °C. Pyroclasts were impregnated with epoxy, cut, and polished for analysis via back-scattered electron (BSE) imaging using a FEI Quanta 200 SEM at the University of Oregon. Images of both the control and the experimental samples permitted assessment of groundmass crystallization caused by heating. BSE images were analyzed with ImageJ software for total heating-induced crystallization by measuring matrix area containing newly crystallized microlites. These measurements should be considered minimum estimates as areas with incipient crystallization (see below) were not included. Experimental run durations, temperatures, and area percent of heating-induced crystallization are listed in supplemental Table 1.

## Results

**Figure 2:**
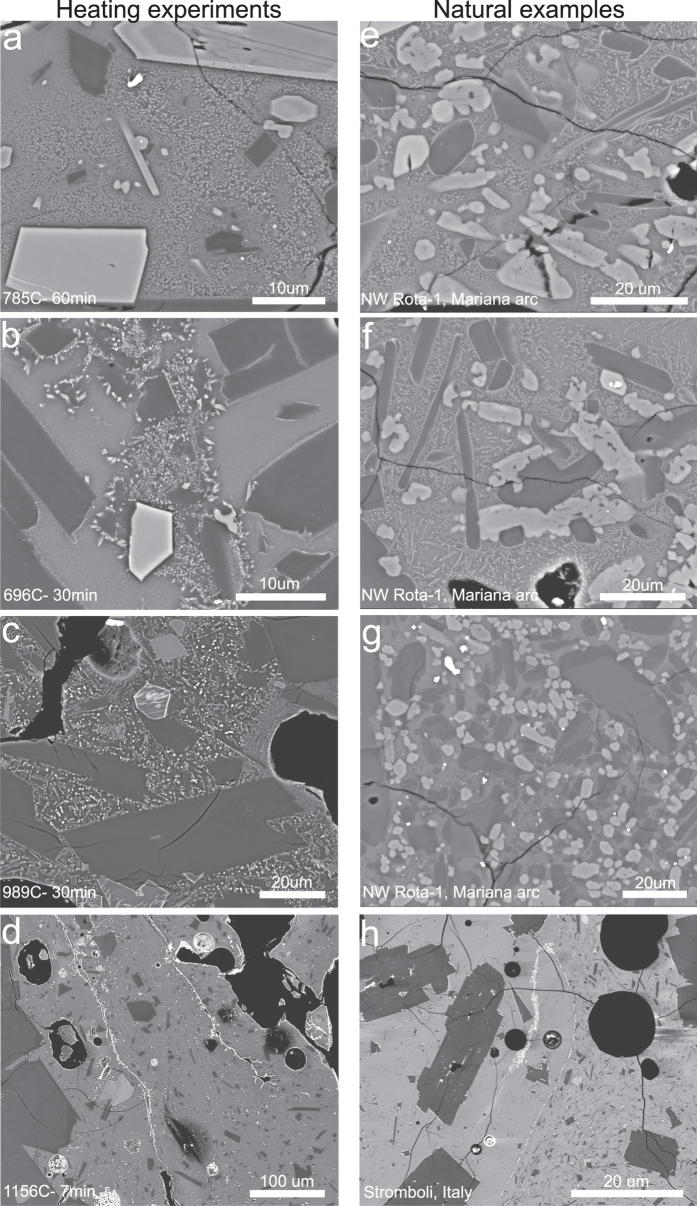
BSE images of experimental crystallization textures (**a–d**) and natural samples with microcrystalline inclusions (**e–h**). (**a,e**) *Incipient crystallization* displaying textures of rapid nucleation and phase separation. (**b,f**) Dendritic growth and *patchy crystallization* between microphenocrysts and on the surfaces of pre-existing crystals. *Extensive crystallization* with no unaffected matrix glass is found in (**c**) and with very little unaffected glass is seen in (**g**). Area around vesicle in (**c**) is altered but lacks extensive crystal growth, likely due to element loss due to diffusion. (**d**) High temperature experiment- glass has begun to flow, collapsing vesicles. Oxides, growing on vesicle and crystal surfaces, form linear features after vesicle collapse. A similar texture is observed in (**h**) where oxides lie along a boundary between microlite-rich and microlite-poor areas.

Heating-induced crystallization textures are illustrated in Fig. 2a–d. The earliest stage of crystallization involves *incipient crystallization*, observed as areas of phase separation (groundmass microlites are just beginning to form, but are not quite recognizable) and the presence of numerous sub-micron-sized crystals (Fig. 2a). As temperature and/or time increase the percentage of newly crystallized matrix increases, transforming from glass to incipiently crystallized areas. A *patchy crystallization* phase follows (Fig. 2b), consisting primarily of localized dendritic growth of pyroxene microlites and expansion of phase separation and nucleation until little or no matrix glass remains (*extensive crystallization*). Dendritic growth is most prevalent in localized areas between plagioclase microphenocrysts; areas that presumably were the first to nucleate (Fig. 2b). *Extensive crystallization* at T ≥ ~800 °C after >60 min and at T > 890 °C after 5 minutes, is characterized by dendritic growth of pyroxenes and oxides and little remaining matrix glass (Fig. 2c). At 900 ≤ T < 1100 °C *three phase extensive crystallization* is observed, where virtually all original glass has transformed to crystals (Fig. 2c). At T > 1100 °C, crystallization of new phases drops abruptly and is *dominated by oxides* (Fig. 2d) that replace pyroxenes as the dominant crystal phase. High temperature experiments (>1150 °C) produced numerous oxides growing in clusters, on microphenocrysts with resorption textures, and on the external surface of the pyroclasts and surfaces of vesicles (likely as sublimates^13^). The high temperature experiments lower the viscosity of the matrix glass (decreasing log viscosity by ~4 Pa s from 690–1150 °C^20^) sufficiently to allow flow, which causes vesicles to collapse, new bubbles to form, and clasts to reshape into fluidal morphologies. The collapsed vesicles form prominent linear oxide strands from oxides formed on vesicle walls (Fig. 2d).

**Figure 3:**
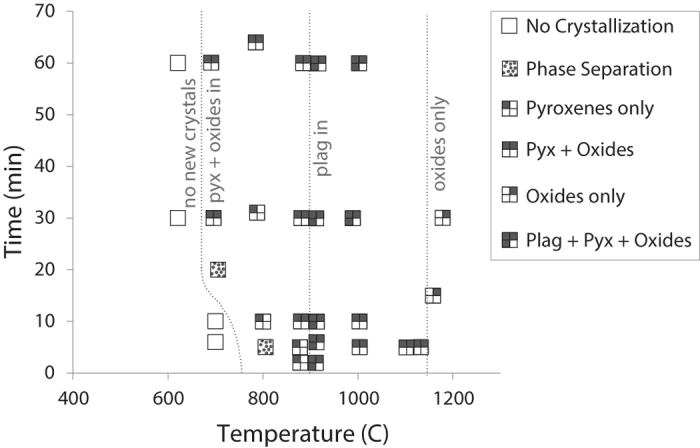
Temperatures and durations of reheating experiments showing mineral phases present after heating. Pyroxenes dominate at low temperatures (<1000 °C); oxides dominate in high temperature experiments (>1000 °C). Plagioclase is observed in experiments of >900 °C. Experiments run at T > 1150 °C show reorganization of glass as viscosity is reduced, allowing flow (i.e. melting).

**Figure 4:**
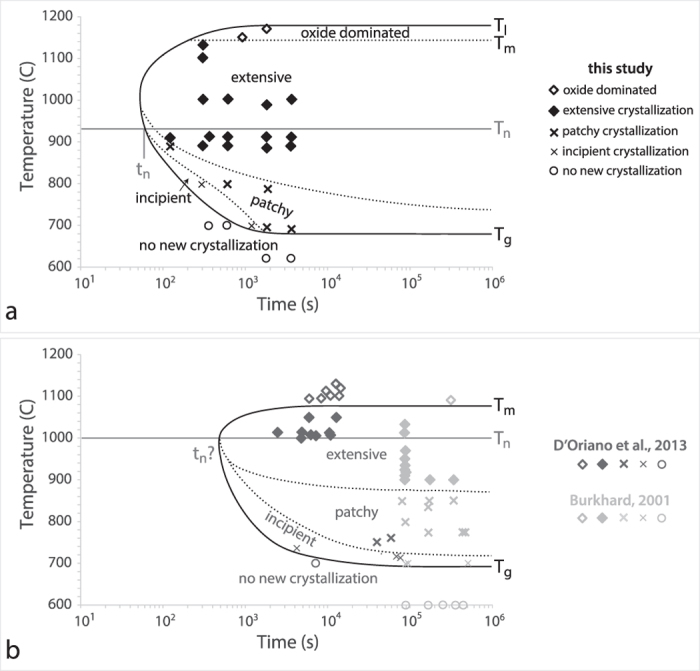
Time-Temperature-Transformation (TTT) diagrams displaying the extent of crystallization observed in reheated tephra from this study (**a**) those of D’Oriano *et al*.^10^,^13^ and Burkhard^14^ (**b**). (**a**) The zones of crystal nucleation and growth are identified by dashed lines. Area percent of new crystallization for each contoured region is as follows: no new crystallization = 0%; incipient crystallization = 2–21%; patchy crystallization = 13–44%; extensive crystallization = 74–100%; oxide dominated = 3–4%. T_n_ represents the average position of the TTT curve in temperature, and the position of the nose in time. Dashed lines in (**b**) approximate crystallization fields from previous experiments^13^,^14^, as determined from sample descriptions. t_n_ for the D’Oriano *et al*.^13^ and Burkhard^14^ samples are unknown. T_g_, T_m_, and T_l_ are estimated temperatures for the glass transition, onset of sample melting, and liquidus, respectively.

Experimental results are summarized in Fig. 3, which shows phase types as a function of temperature and time. Most notable is the temperature dependence of phase appearance, with pyroxene + oxide crystallization at >690 °C followed by plagioclase crystallization at ~900 °C. The apparent absence of oxides in experimental runs of T = ~800 °C and run lengths <60 min, is most likely the result of their small size and the poor atomic number contrast in those BSE images. Plagioclase was observed only at temperatures between 900 °C and 1000 °C, and has characteristic elongate lath and swallowtail morphologies (Fig. 2c). At runs of T = ~1000 °C and ≤10 min, plagioclase was not clearly apparent but may have been present within dark areas between pyroxene microlites. Although clusters of plagioclase microlites are present in high temperature runs (≥1100 °C), the lath and swallowtail rapid growth morphologies observed in experiments between 900 °C and ~1000 °C are not observed. Therefore, we cannot conclusively determine the presence of new plagioclase growth at these temperatures. There is an increase, and then decrease, in total crystallinity as a function of temperature and time, as illustrated in the Time-Temperature-Transformation (TTT) diagram of Fig. 4a. Temperature appears to have the greatest impact on crystallization, illustrated by measurements that show <45% of matrix has crystallized at T ≤ 890 °C, versus >80% new crystallization at 890 °C ≤ T ≤ 990 °C for experimental runs longer than two minutes, and >93% at 990 °C ≤ T ≤ 1132 °C. At T ≥ 1150 °C the extent of new crystallization drops precipitously to 3–4% as only oxides are newly crystallized.

TTT diagrams are used in industrial glass research to constrain critical cooling rates needed to avoid mineral formation. The ‘zone’ of crystallization is indicated by a C-shaped curve (solid black lines in Fig. 4a,b) to the left of which only amorphous glass will form and to the right of which nucleation of mineral phases will occur. These curves are typically determined experimentally, as the location of the curve is dependent on composition and experimental conditions e.g.^21^,^22^,^23^. The average placement of the TTT curve on the temperature axis can be estimated as T_n_ = 0.5(T_g_ + T_l_) e.g.^22^, where T_l_ is the liquidus temperature and T_n_ is the ‘nose’ of the TTT diagram. For our experimental conditions, T_n_ = 934 °C if T_l_ = 1178 °C and T_g_ = 690 °C; we approximate the placement of T_n_ on the time axis using the results of our experiments, which show that the minimum dwelling time (t_n_) for the onset of crystallization for basaltic andesite tephra is ≤120 seconds. T_n_ anchors the TTT curve in Fig. 4a; within the (outer) TTT curve we have added dashed lines to indicate inferred boundaries separating the crystallization fields observed in our experiments.

For comparison, in Fig. 4b we have plotted the experimental data of D’Oriano^13^ and Burkhard^14^, using their sample descriptions. We use a T_n_ of ~1000 °C (after D’Oriano *et al*.^10^), but have fewer constraints on placement of the TTT curve on the time axis because of the long experimental durations (≥40 min for D’Oriano; ≥22 hrs for Burkhard). It should be noted that TTT curves will likely shift due to compositional differences (basaltic andesite, this study; alkali-rich basalts^13^; Kilauea basalt^14^). For example, incipient crystallization was observed at longer timescales in both D’Oriano’s and Burkhard’s experiments (thin Xs) than in those of this study, but at similar temperatures, suggesting the TTT curve of basalt is shifted to the right, with a greater t_n_ than basaltic andesite. More experiments over a greater range of compositions and timescales are needed to explore the exact shapes of TTT heating curves and minimum dwelling times during recycling.

## Discussion

The extent of primary crystallization in basaltic systems is controlled by composition, temperature, pressure, and time, and is driven by cooling and/or decompression. Here we have demonstrated a less intuitive process, which is crystallization by heating glassy samples (often referred to as devitrification), a method contrary to traditional experimental crystallization, which focuses on crystallization as a cooling-driven phenomenon. We further suggest that heating-induced crystallization has been under-appreciated in volcanic environments. Tephra, when rapidly quenched upon interaction with air (or water), is often glassy (meaning it was liquid at the time of eruption). Quenching prior to complete crystallization means that tephra is in a metastable state and requires only an increase in temperature above T_g_ to activate diffusion and initiate (re)crystallization.

Cooling of a basaltic andesite melt at 1 atm would initially induce crystallization of plagioclase, followed by orthopyroxene, clinopyroxene, and finally iron oxides (from MELTS^18^,^19^). In our reheating experiments we see the reverse order, with pyroxenes and oxides the first to crystallize, followed by plagioclase. The mineral sequence and temperatures of crystallization are similar to those found by Burkhard^14^ for Kilauea basalt and D’Oriano *et al*.^13^ for alkali basalt, who covered a more limited temperature range (700–750 °C and 1,000–1,130 °C), but over variable *f*O_2_. These studies suggest the results are due to diffusion, with the first mineral phases to appear having the fastest element diffusion rates, while delayed nucleation of plagioclase requires higher temperatures because of relatively slower diffusion. It is important to note that plagioclase crystallization could also be inhibited if quenched and recycled clasts preserve elevated amounts of H_2_O (e.g. refs 24, 25, 26). Although olivine-hosted melt inclusions from Parícutin volcano record pre-eruption water contents of 1.3–4.2 wt%^5^, we expect the water contents of matrix glass to be very low (~0.1 wt% H_2_O), consistent with the absence of vesiculation upon heating (in contrast to NW Rota-1 samples). Thus although plagioclase suppression has been observed at H_2_O ≤ 0.5 wt%^26^ and could have contributed to suppression of plagioclase at T < 900 °C (and subsequent crystallization if further degassed), we believe variable diffusion rates are most likely to control the crystallization sequence. With increasing temperature, diffusion rates increase, effective supersaturation decreases and the phase assemblage approaches that of the erupted material. That we see continued crystallization of oxides in the high temperature experiments is explained by the high (atmospheric) ƒO_2_ of our experiments, a condition that should also apply to recycling of pyroclasts in subaerial environments.

Our reheating experiments produced a range of crystal textures from localized nucleation of only one or two mineral phases to extensive crystallization with three mineral phases. Many of the experimental crystal textures observed are similar to those found in other experiments^13^,^14^ and are comparable to textures found in microcrystalline inclusions from both submarine and subaerial volcanoes (Fig. 2e–h). *Incipient crystallization* textures (Fig. 2a) were very common in our experimental pyroclasts but were observed in only a couple of NW Rota-1 thin sections (Fig. 2e). Crystal nucleation and additional dendritic growth on pre-existing crystal faces (Fig. 2b) is particularly common, occurring in all experimental runs from T_g_ to >1000 °C, and is present in nearly all microcrystalline inclusions observed in natural samples (Fig. 2f). Extensive crystallization is also common in NW Rota-1 microcrystalline inclusions, which often show three crystal phases (pyroxenes, plagioclase, Fe-sulfides). However, the natural microcrystalline inclusions tend to have unaffected matrix glass between microlites (Fig. 2g); clean glass in extensively crystallized areas of the experimental charges was rare except in very short duration experiments (≤10 min at T = ~890 °C−>1000 °C). This suggests that the recrystallization of microcrystalline inclusions in natural samples likely occurred over very short timescales (<10 min), consistent with the pulsating form of most low-level eruptive activity.

The highest temperature experimental samples show extensive oxide crystallization (Fig. 2d), as also seen in the high *f*O_2_ (atmospheric) experiments of D’Oriano *et al*.^13^. Although we do not see extensive oxide crystallization in the submarine tephra samples from NW Rota-1, we do see these features in subaerial samples from Stromboli volcano (Fig. 2h). Here a thick strand of oxides extending from an oxide border between glassy and crystalline regions resembles linear strands of oxides after vesicle collapse in our reheating experiments (Fig. 2d). The absence of oxides in the submarine samples can thus be explained by the different ƒO_2_ in the submarine environment. While oxidizing conditions are not present in the submarine environment, enrichment of chlorine in microcrystalline inclusions in NW Rota-1 submarine tephra provides evidence of seawater entrainment by recycled clasts^9^. From this we suggest that chlorine assimilation provides a recycling signature in the submarine environment through interaction with seawater^9^, whereas oxide strands may be a characteristic signature of subaerial recycling.

### Conclusions and Implications

The experiments in this study and those of D’Oriano *et al*.^10^,^13^ have shown pyroclasts that fall back into a volcanic vent can experience additional crystallization if heated to temperatures above T_g_. This suggests that in some cases, pyroclasts that exhibit both microlite-poor and microlite-rich textures may record recycling and reheating of previously ejected clasts. This provides an alternative explanation for these textures, and, correspondingly, different implications for eruption conditions. Our experiments also show the onset of crystallization is rapid and temperature dependent, occurring within 20 minutes at T > ~690 °C (T_g_) and within <5 min at T > 800 °C. Moreover, crystallization is extensive when clasts are heated to ≥900 °C. At very high temperatures (≥1150 °C) recycled clasts may also show evidence of deformation, such as flow banding and mingling, as well as vesicle collapse and formation of oxide trails and coatings.

Textures, such as flow banding and mingling, of areas indicating recycling suggest temperatures ≥1150 °C; the temperature at which vesicle collapse and glass flow occurred experimentally under gravity. However, high temperature experiments, near or above the eruption temperature, did not yield the extent of crystallization observed in natural samples. Mingling of microcrystalline inclusions with surrounding matrix may occur at slightly lower temperatures (<1100 °C) due to differential stresses during mixing and churning within the vent.

Recycling of pyroclasts is most likely to occur at volcanoes characterized by mild explosivity and the inability to completely expel all pyroclasts from the vent. Pyroclasts falling back into the vent have the potential to be reheated and recycled, inducing additional degassing and microcrystallization, altering their primary textures that may be used to determine magma ascent, vesiculation, fragmentation, and deposition. The actual extent of pyroclast recycling in the subaerial and submarine environments is unknown, but is likely greatly under-reported. We note, however, that textures consistent with recycling (including high crystallinities and oxide trains or coatings) have been observed at Mt. Etna, Italy^4^,^10^, Stromboli, Italy (this study^2^,^3^), Mt. Vesuvius, Italy^10^, Lava Butte, Oregon Cascades^27^, Parícutin, Mexico^16^, Gaua, Vanuatu^10^, Llaima, Chile^28^. From this we suggest that recycling may be quite common at volcanoes of basaltic to basaltic andesite compositions. Moreover, the higher confining pressure of submarine explosive eruptions should further promote recycling, as suggested by analysis of tephra from the 2006 eruptions of NW Rota-1, Mariana arc, where recycled material comprises up to 15% of the total volume of magma erupted during a single event^9^. However, due to limited sampling and very few observations of submarine eruptions the extent of submarine recycling cannot yet be determined. More extensive study is required to determine the frequency of recycling at individual volcanoes and at low MER volcanoes around the world. As mafic volcanoes are the most abundant on Earth, it is important that we can identify signatures of recycling, along with ‘primary’ textures, in order to correctly interpret eruption dynamics and depositional characteristics. Incorrectly identifying recycling textures could lead to overestimating MERs, and misinterpretation of eruption dynamics and history of crystallization.

## Additional Information

**How to cite this article**: Deardorff, N. and Cashman, K. Rapid crystallization during recycling of basaltic andesite tephra: timescales determined by reheating experiments. *Sci. Rep.* **7**, 46364; doi: 10.1038/srep46364 (2017).

**Publisher's note:** Springer Nature remains neutral with regard to jurisdictional claims in published maps and institutional affiliations.

## Supplementary Material

Supplementary Table 1

## Figures and Tables

**Figure 1:**
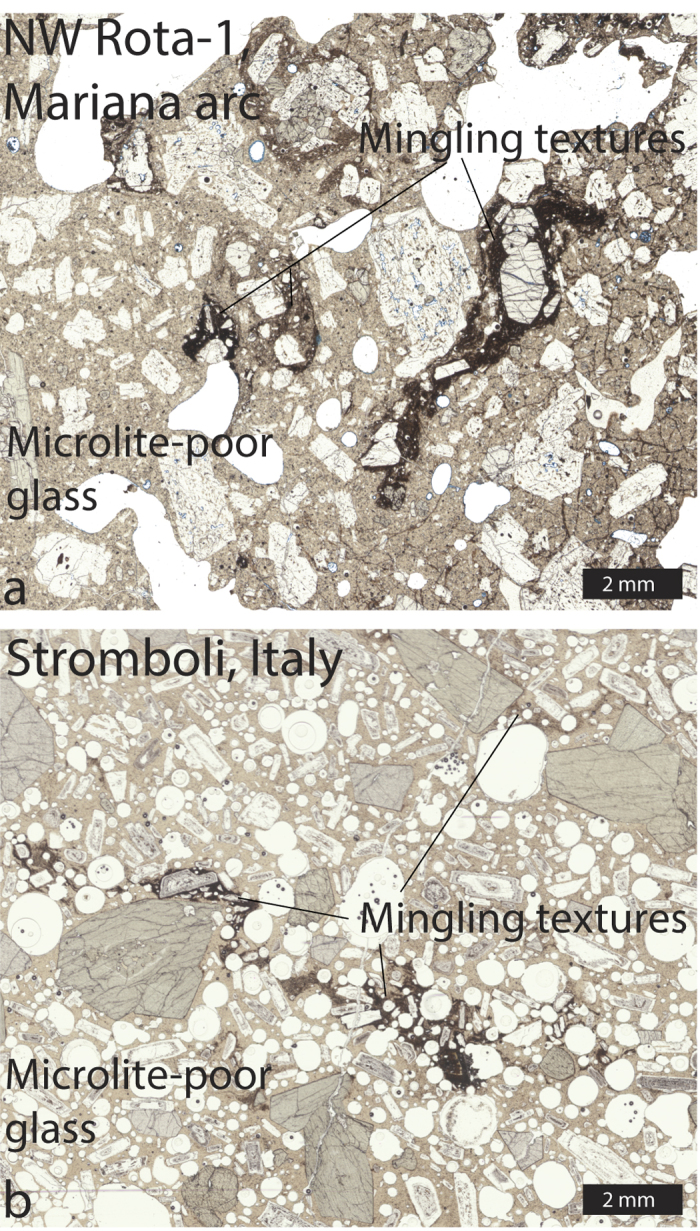
Thin section scans from NW Rota-1 (**a**) and Stromboli (**b**) volcanoes. (**a**) is modified from Deardorff *et al*.^9^. Both thin sections show microlite-poor (tan, sideromelane) matrix glass with microcrystalline (dark brown) inclusions that we interpret to be recycled clasts. Inclusions can have sharp or diffuse boundaries and display textures that suggest mingling with surrounding matrix.
