# Supplementary material for: Rapid crystallization during recycling of basaltic andesite tephra: timescales determined by reheating experiments
Source: Sci Rep. 2017 Apr 12;7:46364. doi: 10.1038/srep46364 (PMC5389347; doi:10.1038/srep46364)
Supplement: Supplementary Table 1 [file srep46364-s1.doc]

**Rapid crystallization during recycling of basaltic andesite tephra: timescales determined by reheating experiments**

| **Sample run** | **Experimental T (°C)** | **Duration (min)** | **% new microlites relative to matrix** |
| --- | --- | --- | --- |
| **Par-S-11** | 603 | 5 | 0.00 |
| **Par-S-7** | 621 | 30 | 0.00 |
| **Par-S-8** | 621 | 60 | -- |
| **Par-S-10** | 691 | 60 | 15.18 |
| **Par-S-10** | 691 | 60 | 17.71 |
| **Par-S-9** | 696 | 30 | 44.43 |
| **Par-S-12** | 700 | 6 | 0.00 |
| **Par-S-13** | 700 | 10 | 0.00 |
| **Par-S-14** | 700 | 20 | 2.15 |
| **Par-S-2** | 785 | 64 | 87.27 |
| **Par-S-1** | 788 | 31 | 12.83 |
| **Par-S-15** | 800 | 10 | 12.87 |
| **Par-S-16** | 800 | 5 | 20.87 |
| **Par-S-24** | 886 | 30 | 100.00 |
| **Par-S-21** | 890 | 5 | 80.43 |
| **Par-S-22** | 890 | 2 | 40.72 |
| **Par-S-23** | 890 | 10 | 97.58 |
| **Par-S-25** | 890 | 60 | 73.99 |
| **Par-S-25** | 890 | 60 | 94.60 |
| **Par-S-29** | 911 | 60 | 85.47 |
| **Par-S-30** | 911 | 2 | 94.34 |
| **Par-S-31** | 911 | 2 | -- |
| **Par-S-28** | 912 | 30 | 100.00 |
| **Par-S-26** | 913 | 6 | 97.32 |
| **Par-S-27** | 913 | 10 | 88.20 |
| **Par-S-3** | 989 | 30 | 100.00 |
| **Par-S-4** | 1002 | 60 | 100.00 |
| **Par-S-17** | 1003 | 5 | 93.31 |
| **Par-S-18** | 1003 | 10 | 100.00 |
| **Par-S-19** | 1102 | 5 | 100.00 |
| **Par-S-20** | 1132 | 5 | 95.23 |
| **Par-S-6** | 1151 | 15 | 3.30 |
| **Par-S-5** | 1171 | 30 | 3.78 |

**Nicholas Deardorff** and **Katharine Cashman**
